# CancerOmicsNet: a multi-omics network-based approach to anti-cancer drug profiling

**DOI:** 10.18632/oncotarget.28234

**Published:** 2022-05-19

**Authors:** Limeng Pu, Manali Singha, Jagannathan Ramanujam, Michal Brylinski

**Affiliations:** ^1^Center for Computation and Technology, Louisiana State University, Baton Rouge, LA 70803, USA; ^2^Department of Biological Sciences, Louisiana State University, Baton Rouge, LA 70803, USA; ^3^Division of Electrical and Computer Engineering, Louisiana State University, Baton Rouge, LA 70803, USA; ^*^These authors contributed equally to this work

**Keywords:** cancer growth rate, kinase inhibitors, differential gene expression, gene-disease association, cancer-specific networks

## Abstract

Development of novel anti-cancer treatments requires not only a comprehensive knowledge of cancer processes and drug mechanisms of action, but also the ability to accurately predict the response of various cancer cell lines to therapeutics. Numerous computational methods have been developed to address this issue, including algorithms employing supervised machine learning. Nonetheless, high prediction accuracies reported for many of these techniques may result from a significant overlap among training, validation, and testing sets, making existing predictors inapplicable to new data. To address these issues, we developed CancerOmicsNet, a graph neural network with sophisticated attention propagation mechanisms to predict the therapeutic effects of kinase inhibitors across various tumors. Emphasizing on the system-level complexity of cancer, CancerOmicsNet integrates multiple heterogeneous data, such as biological networks, genomics, inhibitor profiling, and gene-disease associations, into a unified graph structure. The performance of CancerOmicsNet, properly cross-validated at the tissue level, is 0.83 in terms of the area under the receiver operating characteristics, which is notably higher than those measured for other approaches. CancerOmicsNet generalizes well to unseen data, i.e., it can predict therapeutic effects across a variety of cancer cell lines and inhibitors. CancerOmicsNet is freely available to the academic community at https://github.com/pulimeng/CancerOmicsNet.

## INTRODUCTION

Cancer is perhaps best understood as a complex system of interacting molecular-level networks, such as nuclear and cell networks, influenced by local and distant factors [1]. The nuclear network is composed of nucleic acid and protein molecules linked by a variety of biochemical and structural pathways allowing for the production of proteins based on the information encoded in the DNA [2]. Numerous curative cancer treatments have been developed either by targeting a single component within this network or by combining multiple agents to target different levels of the nuclear network in order to interrupt nucleic acid and protein machineries in the nucleus [3]. The cell network consists of various molecules interacting through the linkage of signal transduction pathways and the cytoskeleton [4, 5]. Particularly, the modulation of the activity of receptor tyrosine kinases, important components of the cell network, is an effective strategy against a wide variety of cancers [6]. This therapeutic effect can be achieved by either blocking upstream receptors with antibodies and small molecules or directly suppressing kinase catalytic activity with inhibitors [7]. Another group of therapies targeting the cell network disrupt metabolism by affecting the function of proteasome and chaperone molecules [8]. Since many cancer-specific data, such as molecular interactions, belong to the non-Euclidean space, a network-based representation of cancer is generally well suited not only to predict the response of tumor cells to pharmacotherapy, but also to help understand drug-cell line interactions. However, utilizing these information-rich data requires advanced graph information processing algorithms and machine learning systems designed specifically to operate on the graph-structured data.

One of the earliest graph information processing techniques is a graph neural network (GNN) that employs a graph structure to learn the representation of the input data [9]. The major limitation of this method is that it restricts the information propagation to the first-order neighbors of every node limiting the information flow in the model. Recently, a graph convolutional network (GCN) was proposed to provide a more flexible model propagating information through many orders of neighbors [10, 11]. More advanced models were developed following the fundamental work on GCN, including a graph-based neural network employing the long-short term memory (LSTM) to carry out the information propagation that was demonstrated to have a significantly improved performance [12]. Another information propagation scheme aggregates the average embeddings of the neighboring nodes yielding a high performance especially for node classification in large graphs [13]. Numerous other techniques implementing minor improvements are currently available to operate on the graph-structured data [14–16].

Compared to other types of biological networks, gene co-expression networks have certain advantages, such as a high coverage of human genes, the additional knowledge obtained from the biomedical literature, and the possibility to study different cancer subtypes. [17, 18]. One of the most important applications of gene co-expression networks is to study the sensitivity of cancer cells to pharmacotherapy. Indeed, networks constructed by connecting those genes having correlated drug-induced expression values, contain a sufficient amount of information to predict drug sensitivity. In a recent study, two feature selection methods, network- and correlation-based, were developed to extract representative features for drug response prediction from gene co-expression networks [19]. The network-based feature selection utilizes assignment vectors describing the importance of individual vertices to predict drug sensitivity, whereas the correlation-based selection employs the Pearson correlation coefficient (PCC) between gene expression and the sensitivity of cell lines to drugs. Benchmarking calculations against non-small cell lung cancer with several canonical prediction algorithms, Elastic Net, Partial Least Squares Regression, Random Forest, Support Vector Regression, and Deep Neural Networks, demonstrated that features extracted with the network-based approach yield the highest performance when predicting the dose-response curve and the median effective dose.

Another group of methods utilize dual-layer cell line-drug networks, constructed by integrating drug similarity and cell line similarity networks in a weighted fashion, to predict the drug sensitivity of cancer cells. These techniques build on the observation that chemically similar drugs exhibit similar inhibitory effects on different cell lines and vice versa, similar cell lines tend to respond comparably to a treatment with the same drug. Dual-layer models typically require the optimization of various parameters, such as weights for individual drugs and cell lines, in order to determine the relative contribution of each network to the final prediction. As an example, a dual-layer network was developed to evaluate separately the response of a known cell line to a new drug and the effect of a known drug against a new cell line using a linear weighted model, followed by combining these two quantities into a sensitivity score for the treatment of a particular cell line with a drug [20]. Encouragingly, comprehensive benchmarks against the Cancer Cell Line Encyclopedia (CCLE) [21] and the Cancer Genome Project (CGP) [22] datasets showed that the predicted and observed therapeutic responses are correlated for most tested drugs with a PCC of 0.6, significantly outperforming an Elastic Net model. Additionally, this dual-layer integrated cell line-drug network model correctly predicted that certain mutant cell lines are more sensitive to inhibitors than the corresponding wild-type cell lines even though no mutation-specific information was provided.

More advanced methods combine genomics with drug chemical and activity information to predict the response to drugs in cancer treatment. For instance, the Cancer Drug Response Profile scan, or CDRscan, predicts anticancer drug responsiveness based on the drug screening assay data, the genomic profiles of human cancer cell lines, and the molecular fingerprints of drugs [23]. The analysis of observed and predicted drug responses showed an exceptionally high accuracy of CDRscan with a mean coefficient of determination of 0.84 and the area under the receiver operating characteristics (ROC) of 0.98. Another technique, DeepDR, predicts drug response purely based on the mutation and expression profiles of cancer cells. The reported overall prediction performance of DeepDR is also exceptionally high with a mean squared error of only 1.96 in the log-scale IC_50_ values. Further, a similarity-regularized matrix factorization method, or SRMF, predicts anticancer drug responses of cell lines solely from the chemical structures of drugs and the baseline gene expression levels in cell lines [24]. Those two features are used as regularization terms, which are incorporated into the drug response matrix factorization model. SRMF yields a drug-averaged mean squared error of 1.73 between predicted and observed responses of sensitive and resistant cell lines.

Notwithstanding these encouraging reports, there are two drawbacks of currently available techniques to predict the response of cancer to drug treatment. First, most of these methods employ hand-crafted features simply exploiting similarities between instances, i.e., they essentially look for similar combinations of cell lines and drugs with known therapeutic outcomes. In reality, similar cell line-drug combinations may not necessarily produce the anticipated effects. Explicit similarity-based approaches are also unlikely to reveal the underlying mechanisms of the response of cancer to drug treatment. Second, the performance of many existing algorithms is likely grossly overestimated due to randomly splitting the redundant data into training, validation, and testing subsets resulting in a significant overlap among these sets. To address both issues, we developed CancerOmicsNet, a GNN-based algorithm employing multiple graph convolutional blocks with the attention-based propagation and a sophisticated graph readout mechanism to predict the effect of a drug treatment on the cancer cell growth. This novel method utilizes compact, cancer-specific networks constructed from protein-protein interactions, differential gene expression, disease-gene association, and drug inhibition data. The generalizability of CancerOmicsNet is carefully evaluated in a series of cross-validation benchmarks against different tumor tissues.

## RESULTS

### Cancer-specific data represented as networks

**Figure 1:**
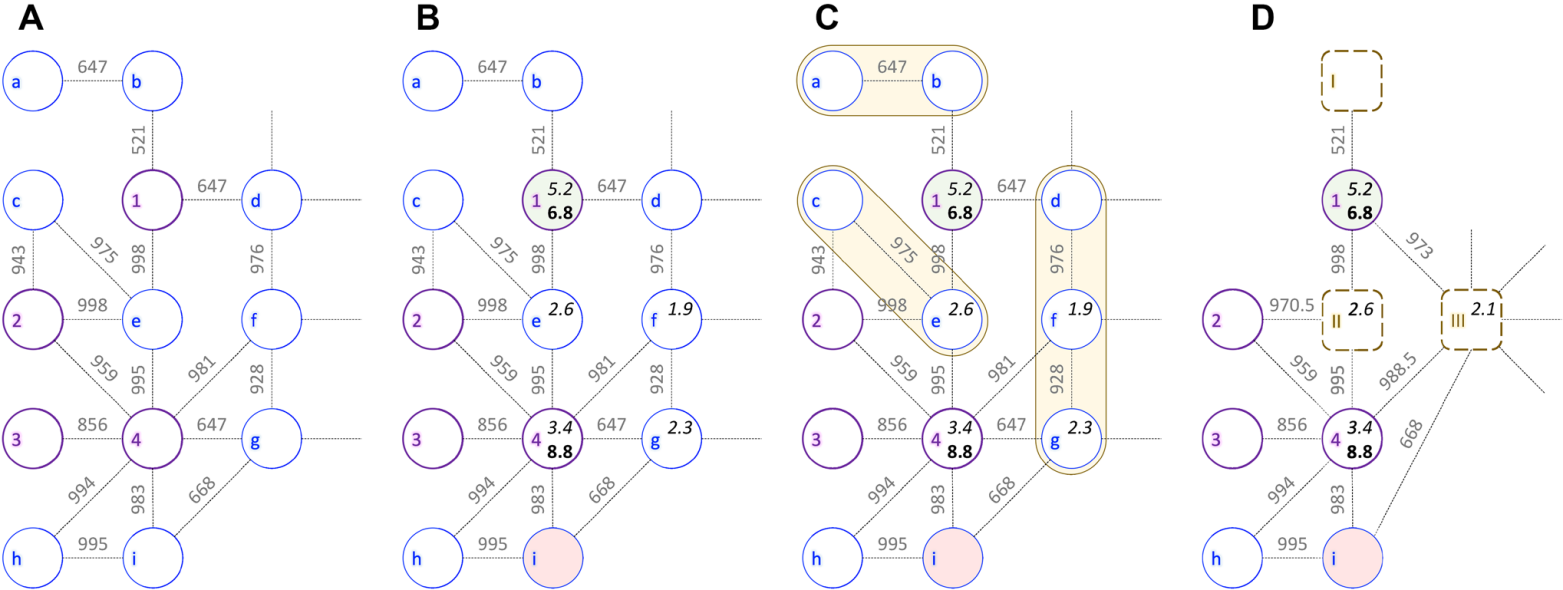
Example of a cancer-specific subnetwork. The graph shows a portion of protein-protein interaction network for breast adenocarcinoma cell line MDA-MB-468 and kinase inhibitor dasatinib. Bold purple circles represent kinase nodes (1 – EGFR, 2 – JAK2, 3 – JAK1, and 4 – SRC), whereas non-kinase nodes are shown as thin blue circles (a – NANOG, b – EP300, c – IL10RA, d – HIF1A, e – STAT3, f – HSP90AA1, g – PXN, h – CRK, and i – CBL). Edge weights are confidence scores for protein-protein interactions with a threshold value of ≥500. (**A**) Initial subnetwork constructed from interactions obtained from the STRING database. (**B**) Subnetwork integrating kinase inhibitor profiling (pIC_50_, in bold), disease-gene association scores (in italics), and the differential gene expression: up- (green), down- (red), and normally (gray) regulated. (**C**) Graph reduction procedure with orange shapes outlining groups of non-kinase nodes that have similar differential gene expression and belong to the same GOGO cluster. (**D**) Reduced cancer-specific subnetwork with merged nodes shown as dashed brown rounded boxes (I – constructed from incident nodes a-b, II – c-e, and III – d-f-g). Node features and edge weights for merged nodes are calculated as median values of incident nodes.

Input for CancerOmicsNet are cancer-specific networks assembled from multiple heterogeneous data including protein-protein interactions (PPIs), differential gene expression (DGE), disease-gene association (DGA) scores, kinase inhibitor profiling (KIP), and growth rate inhibition (GR). The procedure of data integration is schematically presented in Figure 1 for a combination of breast adenocarcinoma cell line MDA-MB-468 originated from a 51 years old female sample [25], and dasatinib, a dual kinase inhibitor against BCR/ABL and SRC families of tyrosine kinases [26] primarily used to treat chronic myelogenous leukemia and acute lymphoblastic leukemia [27]. In this example subnetwork, nodes (circles) are proteins and dashed lines represent highly confident PPIs. Bold purple circles are kinase nodes and thin blue circles are non-kinase proteins.

After the initial network is constructed (Figure 1A), proteins are annotated with DGE, DGA, and KIP scores (Figure 1B). EGFR is a transmembrane receptor tyrosine kinase having a critical impact on the regulation of apoptosis, cell migration, and cell proliferation. Since it is hyper-expressed in MDA-MB-468 cell line [28], node 1 in Figure 1B is colored green. On the other hand, node i is colored red because ubiquitin ligase CBL is deregulated in breast cancer [29]. In normal cells, CBL mediated ubiquitination negatively regulates EGFR by lysosomal degradation [30], however, CBL mutants escape the degradation of overexpressed EGFR inducing oncogenesis [29]. Next, DGA data for MDA-MB-468 cell line are mapped to proteins in the network; kinase nodes 1 and 4 are assigned DGA scores of 5.2 and 3.4, whereas non-kinase proteins e, f, and g have DGA scores of 2.6, 1.9, and 2.3, respectively. EGFR has the highest DGA score for breast adenocarcinoma likely because it is hyper-expressed in approximately half of the cases of inflammatory breast cancer and triple-negative breast cancer [31].

Subsequently, the inhibition data against dasatinib are added to the network. Dasatinib inhibits SRC with an IC_50_ value of 0.8 nM in a cell-free assay [32] and different variants of EGFR with IC_50_ ranging from 21.7 to 138 nM [33]. Two kinase nodes (1 and 3) are annotated with pIC_50_ values for dasatinib (6.8 and 8.8). Finally, the entire graph is assigned a label describing the effectiveness of the drug therapy against a given cell line. Since the growth of MDA-MB-468 cell line is inhibited by 30% 48 hrs after the treatment with dasatinib at 3 μm [34] and the experimental GR_max_ value [35] is –0.96, the label of the MDA-MB-468-dasatinib combination is a positive pharmacotherapeutic effect.

### Network reduction driven by biological knowledge

Cancer-specific networks are subsequently subjected to a reduction procedure devised to produce graphs that are more compact yet richer in the biological information. This algorithm is presented in Figure 1C for the MDA-MB-468-dasatinib subnetwork. Briefly, a group of connected non-kinase proteins having similar DGE values and being part of the same biological processes according to Gene Ontology [36] are merged into a single node. Three such groups are present in the example subnetwork, a-b, c-e, and d-f-g (yellow shapes in Figure 1C). The first group comprises transcription factor P300, a product of EP300 gene, regulating the expression of NANOG that is responsible for pluripotency and self-renewal of stem cells [37]. The second group consists of a transcription activator STAT3 regulating the expression of IL10 [38]. The last cluster contains HSP90AA1 and HIF1A that together regulate the oxygen homeostasis [39] and PXN, a multidomain and multifunctional focal adhesion adaptor protein playing an essential role in the oxidative stress in cells [40]. The resulting virtual nodes in the reduced graph (dashed rounded squares in Figure 1D) representing multiple proteins involved in the same biological processes have a similar expression in cancer cells and are annotated with a median value of the DGE scores of incident nodes.

### Information propagation in CancerOmicsNet

**Figure 2:**
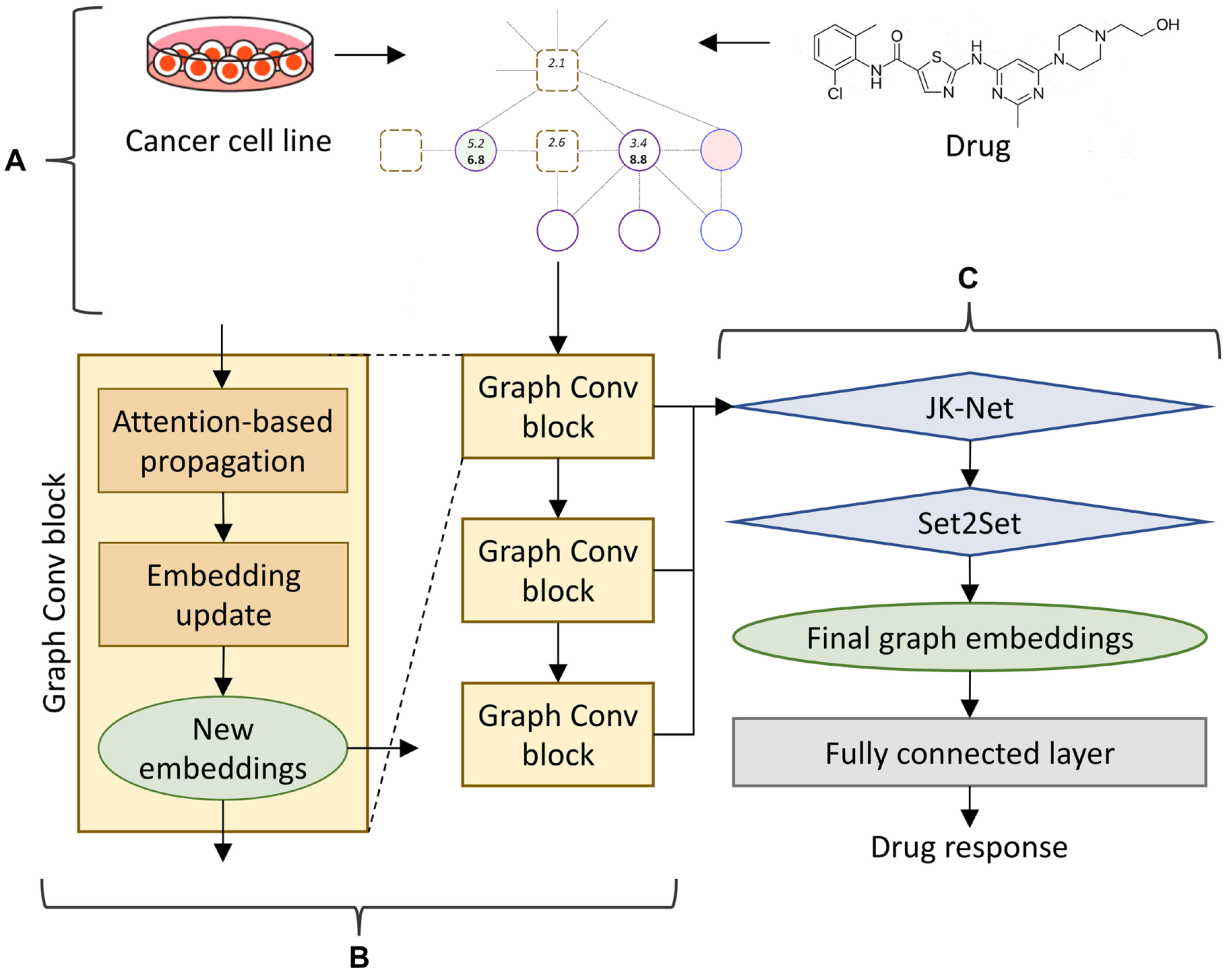
CancerOmicsNet architecture. (**A**) The input is a reduced graph constructed for the combination of a cell line and a small molecule inhibitor. (**B**) The graph is processed through a cascade of three graph convolutional blocks. Within each block, an attention-based propagation is first utilized to pass the information among nodes, and then a graph isomorphism network is employed to update the embeddings for each node. (**C**) Node embeddings generated by all blocks in B are combined using a JK-Net layer and passed to a Set2Set pooling layer serving as the read-out function to acquire the final graph embeddings. At the end, graph embeddings are sent to a fully connected layer to predict the drug response.

CancerOmicsNet implements a GNN model to predict the response of cancer cell lines to a treatment with kinase inhibitors. The GNN employs graph convolutions, which are functionally equivalent to matrix convolutions in the convolutional neural network (CNN) working with images. Similar to the CNN propagating the information of a pixel to its neighbor pixels, the GNN propagates the information of a node in the graph to its neighbor nodes. The architecture of CancerOmicsNet is presented in Figure 2. An instance consisting of the combination of a cell line and a drug is used to create a cancer-specific network, which is subsequently subjected to the reduction procedure (Figure 2A). The reduced graph is then processed through a cascade of graph convolution blocks (Figure 2B). Each block contains three components, the attention-based propagation, the embedding update, and the generation of new embeddings. Although only the information from 1^st^ order neighbors is passed between nodes in a single block, using multiple sequential blocks propagates the information from higher order neighbors.

**Figure 3:**
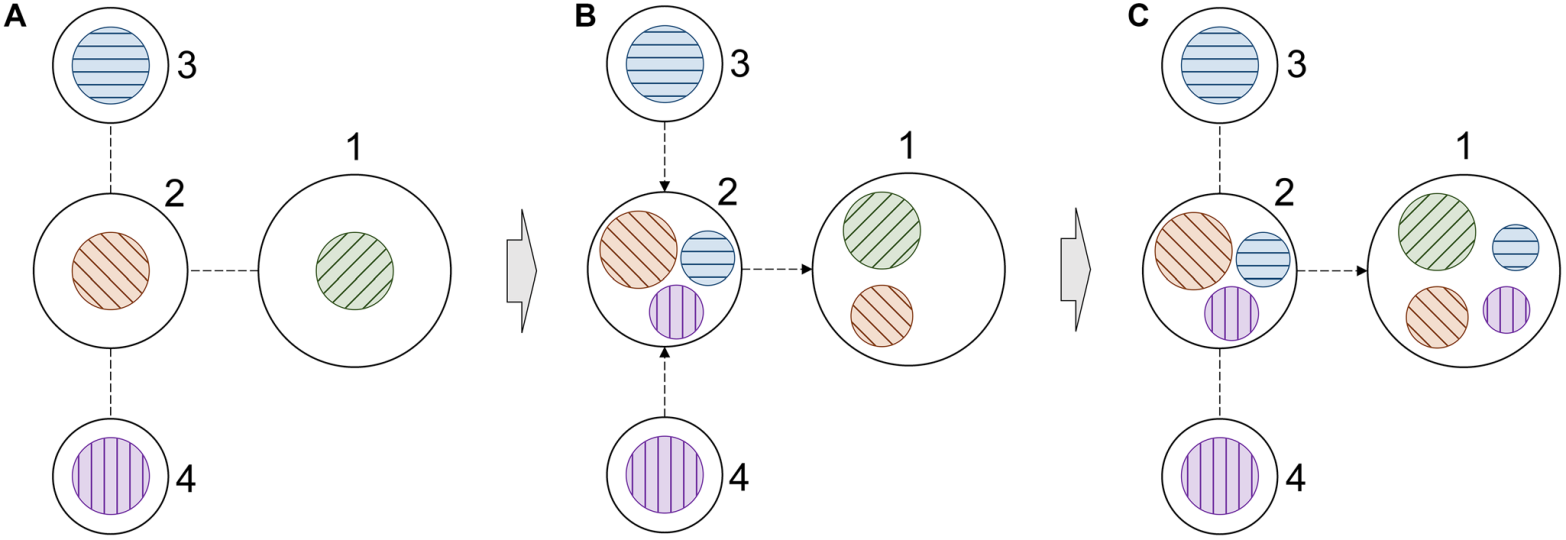
Schematic of information propagation in a graph. (**A**) A simple 4-node graph, in which each node contains its own information. The information is color coded, node 1 – green, node 2 – orange, node 3 – blue, and node 4 – purple. (**B**) The distribution of information within the graph after the first propagation step. (**C**) The distribution of information within the graph after the second propagation step. Only the information propagation to node 1 is illustrated in order to demonstrate how it receives information from higher order neighbors.

This procedure is illustrated in Figure 3 for a simple 4-node graph. Initially, each node has its own information (color coded in Figure 3A), which is used to generate node embeddings. In our model, nodes are proteins connected through PPIs and the information comprises DGE, DGA, and KIP. During the first propagation step, a node of interest, such as node 1 in Figure 3, receives information from its 1^st^ order neighbor, node 2 (Figure 3B). At the same time, node 2 receives information from its 1^st^ order neighbors, nodes 3 and 4. Nodes 1 and 2 now contain more information to generate new embeddings. In the second propagation step, the information from nodes 3 and 4 already present in node 2 is also passed to node 1 (Figure 3C). At this point, new embeddings for node 1 are generated using not only its own information, but also the information propagated from its 1^st^ and 2^nd^ order neighbors. Three graph convolution blocks are employed in our model because we found empirically that adding the fourth block does not improve the performance anymore. Further, there is no point of using more than four blocks because the diameter of the cancer-specific graph is 5, so no new information is propagated beyond 4^th^ order neighbors.

### Graph information extraction

Once all embeddings are generated, the information on the entire graph can be extracted with a readout mechanism to predict the final drug response (Figure 2C). Standard readout techniques, such as global pooling, are unsuitable for our model comprising multiple graph convolutional blocks and learning from highly heterogeneous input graphs. In CancerOmicsNet, node embeddings generated by consecutive graph convolutional blocks contain distinct information. Therefore, a jumping knowledge network (JK-Net) is employed to exploit all information collected from different blocks. JK-Net was specifically developed to efficiently integrate the output from different layers into a single representation [41]. It is based on the concept of an influence radius corresponding to the radius of neighbors whose output is to be aggregated. The selection of an optimal radius is crucial because large radii may cause excessive averaging and small radii may result in an insufficient information aggregation. JK-Net learns the effective neighborhood size for each layer in order to generate the best representation of the entire graph.

Global pooling of the embeddings of all nodes is appropriate only for homogeneous networks. In contrast, cancer-specific networks are highly heterogeneous comprising nodes of varying importance to one another and to the overall graph. Therefore, we added a mechanism to emphasize on important nodes rather than treating all nodes equally. Although such techniques have successfully been used in the CNN and the RNN [42], unlike images or text, graphs are orderless, i.e. an image does not remain the same if pixels are rearranged, while a graph remains the same if nodes are reordered. To account for the lack of order in graphs, we added a Set2Set layer converting a set to another set [43]. This model employs a set of LSTMs recursively combining the state of the previous processing step with the current embeddings to generate attention values. These attention values and embeddings form new states for the next processing step. By using Set2Set, we ensure that any permutation performed on the original vector does not affect the final read vector. The information summarized by JK-Net and Set2Set for the entire graph is then passed to a set of fully connected layers to make the final prediction, which is the effect of pharmacotherapy on the cancer cell growth.

### Performance of CancerOmicsNet compared to other methods

**Figure 4:**
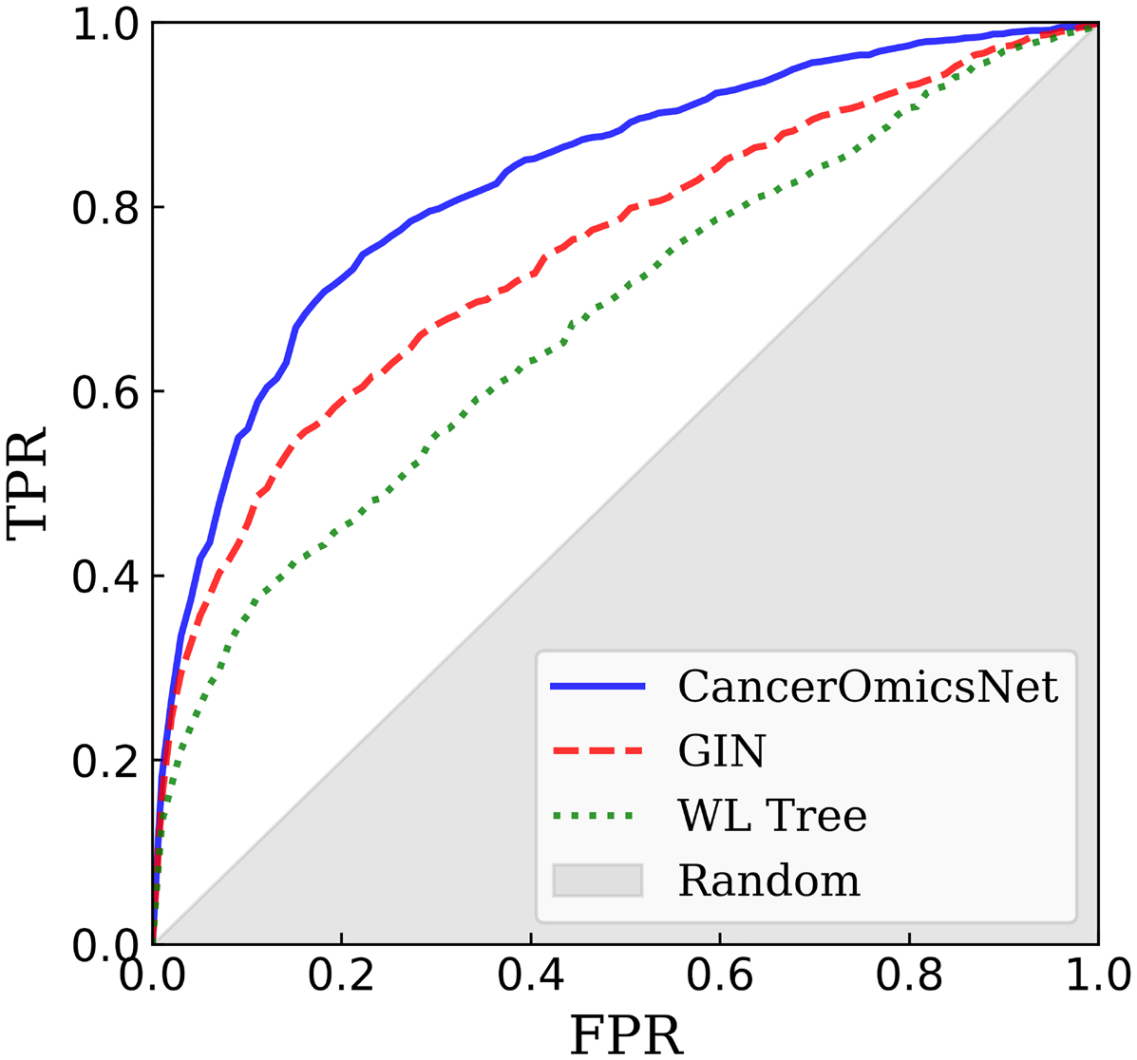
Performance of graph-based algorithms to predict the response of cancer cell lines to drugs. The performance of each method is cross-validated at the tissue level. CancerOmicsNet (solid blue line) is compared to the graph isomorphism network (GIN, dashed red line) utilizing equal propagation, and WL Tree (dotted green line) employing the Weisfeiler-Lehman graph kernel. TPR is the true positive rate, FPR is the false positive rate, and the gray area corresponds to the performance of a random predictor.

In order to properly evaluate the generalizability of CancerOmicsNet, we performed a cross-validation at the tissue level. The entire dataset was first divided into nine groups of different tissues, digestive system, respiratory system, haematopoietic and lymphoid tissue, breast tissue, female reproductive system, skin, nervous system, excretory system, and others. Next, we conducted a 9-fold cross-validation, each time using cancer cell lines from one tissue as a validation set while the remaining cancer cell lines were used for model training. Since cell lines collected from different tissues have different gene expression patterns, this cross-validation scheme eliminates the overlap between training and validation data because the reduced graphs have different topologies. In addition, there is also a desired variability in feature matrices on account of different gene-disease associations, which depend on the cell line and tissue type. Essentially, each fold has entirely different training and validation data. Figure 4 shows a cross-validated ROC plot for CancerOmicsNet compared to other graph-based methods. Indeed, CancerOmicsNet not only gives the highest area under the curve (AUC) of 0.83 ± 0.02, but the AUC values do not vary significantly for different tissues, digestive system (0.85), respiratory system (0.80), haematopoietic and lymphoid tissue (0.81), breast tissue (0.82), female reproductive system (0.86), skin (0.85), nervous system (0.83), excretory system (0.83), and others (0.81).

Removing the attention mechanism, which detects important nodes and puts more weight on them (labeled as GIN in Figure 4), decreases the AUC to 0.75 ±0.04 demonstrating that the propagation attention is an important component of CancerOmicsNet. Further, the performance of CancerOmicsNet is compared to that of the Weisfeiler-Lehman (WL) Tree [44]. Not only the AUC for WL Tree of 0.68 ±0.03 is lower than that for CancerOmicsNet, but since WL Tree processes one graph at a time, its runtimes are much longer than those for CancerOmicsNet featuring batch processing. Finally, Table 1 reports several performance metrics for two deep learning-based methods, CancerOmicsNet and CDRScan [23]. The precision quantifies the number of positive class predictions actually belonging to the positive class, whereas the recall quantifies the number of positive class predictions made out of all positive examples in the dataset. The balanced accuracy is computed as the average recall over all classes to addresses the imbalanced dataset problem [45]. The F-measure provides a single score balancing the concerns of both precision and recall in one number [46]. Encouragingly, using CancerOmicsNet yields up to 14% performance improvement over CDRScan. Overall, these results demonstrate that CancerOmicsNet outperforms other graph kernel and deep learning approaches.

**Table 1:** Performance of CancerOmicsNet and CDRScan in predicting the response of cancer cell lines to drugs

Method	Balanced accuracy	Precision	Recall	F-measure
CancerOmicsNet	0.781	0.764	0.770	0.766
CDRScan	0.637	0.711	0.637	0.632

Accuracy, precision, recall, and F-score are calculated based on the cross-validation at the tissue level.

## DISCUSSION

In this study, we developed CancerOmicsNet, a graph neural network model to predict the growth rate of a cancer cell line after drug treatment. CancerOmicsNet is more advanced than many deep learning techniques operating in the Euclidean space [47], because it extracts knowledge directly from biological networks providing a more adequate representation of complex diseases such as cancer. Further, we implemented a sophisticated attention mechanism to propagate information more efficiently from the most important nodes in the graph when generating node embeddings. Attention mechanisms assigning trainable weights to nodes during information propagation are used to improve not only the classification performance [48, 49], but also the capability to generalize to larger, more complex, and noisy graphs [50, 51]. In our case, this technique allows the GNN model to direct more attention to kinase nodes since many of them contain valuable information on differential gene expression and the level of inhibition by small molecules across different cancer cell lines. As a result, the GNN achieves a better performance, especially against highly heterogeneous networks, such as cancer-specific networks employed in this study.

In order to evaluate the performance of CancerOmicsNet, we conducted a cross-validation at the tissue level by removing from model training all cell lines originating from a particular tissue and then analyzing the accuracy for these cell lines. We put a special attention to design a proper benchmarking protocol since in the context of predictive models, misunderstanding cross-validation very often yields an impressive, yet grossly overestimated predictor performance [52]. Numerous examples of exaggerated results in biomedical studies due to a problematic cross-validation include cancer prediction [53], the prediction of cancer cell line sensitivity and compound potency [54], the identification of drug-target interactions [55], the prediction of optimal drug therapies [56], the estimation of drug-target binding affinities [57], and virtual screening [58]. Since multiple instances in our dataset share cell lines originating from the same tissue, employing cross-validation at the tissue level is critical because splitting the dataset randomly into folds would cause training and validation instances to have a significant overlap with respect to graph topology as well as certain features such as gene expression and gene-disease associations. Encouragingly, the cross-validated accuracy of CancerOmicsNet at the tissue level is significantly higher than those measured for other approaches on the same data. Nonetheless, we note that the applicability of CancerOmicsNet is at present limited to kinase inhibitors, while alternative methods are applicable to other classes of therapeutics as well. Overall, CancerOmicsNet offers a high performance and the desired generalizability in the prediction of the effect of kinase-targeted therapies on the cancer cell growth.

## MATERIALS AND METHODS

### Cancer-specific molecular networks

Input graphs are constructed by mapping multiple heterogeneous data, DGE, KIP, DGA, and GR, on the human PPI network. STRING v11 database [59] has been used to construct the PPI network with an edge confidence threshold of ≥500. The resulting network comprises 19,144 proteins and 685,198 interactions. The DGE data were obtained from the curated Cancer Cell Line Encyclopedia (CCLE) containing the information on normally, up- and down-regulated genes for 749,551 associations between 18,022 genes and 1,035 cancer cell lines [21]. The KIP data on the half maximal inhibitory concentration (IC_50_) for 49,348 small molecules and 411 kinases were collected from Team-SKI [60] and filtered at a minimum threshold of pIC_50_ (the negative logarithm of IC_50_) of 6.3, which is equivalent to 500 nM in terms of IC_50_. The DGA data were obtained from the DISEASE database [61] of 8,330 diseases and 20,715 genes, and the DisGeNET database [62] of 24,166 diseases and 17,545 genes. The association scores range from 1 to 10 in DISEASE and from 0.01 to 1 in DisGeNET databases.

### Growth rate inhibition data

Recent drug response metrics, GR_50_ and GR_max_, quantify the proliferation with the value of growth rate inhibition (GR) based on time course and endpoint assays [35]. GR_50_ is the concentration of a drug at which GR is 0.5, whereas GR_max_ is the maximum measured GR value. Negative GR_max_ values correspond to the cytotoxic response and positive values correspond to the cytostatic response. In this study, we employ six LINCS-Dose-Response datasets, Broad-HMS LINCS Joint Project, LINCS MCF10A Common Project, HMS LINCS Seeding Density Project, MEP-HMS LINCS Joint Project, Genentech Cell Line Screening Initiative, and Cancer Therapeutics Response Portal [35]. The original dataset contains 632 cell lines from different cancer tissues and 795 small molecules tested against those cancer cell lines, totaling 83,162 combinations. After mapping the GR data to the constructed cancer-specific molecular networks and removing those cases having either GR_50_ values set to infinity or multiple GR_50_ values for a particular cell line-drug combination, the final dataset comprises 359 cell lines, 29 drugs, and 3,549 cell line-drug combinations. The number of positive instances (the cytotoxic effects of drugs on cell lines) is 2,124, whereas the number of negative instances (cytostatic responses) is 1,425.

### Graph reduction

A procedure devised to reduce the size of drug-cell line networks employs the topological information and the biological knowledge. Two neighboring nodes are merged when the following conditions are met, both nodes are kinase proteins, share the same gene expression, and belong to the same GOGO [63] cluster representing proteins involved in similar biological processes. Applying the graph reduction procedure produces smaller graphs with the average number of nodes of 1,349 and the average number of edges of 12,613. Even though the graph sizes are significantly reduced by more than 90%, the percentage of kinase nodes carrying most of the meaningful information increases from 2% to 30%. Another advantage of reduced graphs over full-size networks is their topological diversity created by differences in the gene expression profiles of various cancer cell lines.

### Information propagation

The most widely adopted propagation protocol transmit the information equally without considering the importance of a node to its neighbors and to the graph. This protocol can be expressed as

$$\begin{array}{cc} X^{\left(t\right)}=D^{-1/2}AD^{-1/2}X^{\left(t-1\right)} & \text{Equation 1} \end{array}$$

where *t* is the propagation step, *D* is the degree matrix of the adjacency matrix *A*, and *X*^(t)^ represents embeddings at the propagation step *t*. Note that the original node features can be denoted as the 0-th propagation step, *X*^(0)^. It is obvious that not all nodes have the same importance to their neighbors. For instance, many non-kinases in our dataset contain no useful information because these proteins are normally expressed, have no association with a disease, and are not targets for inhibitors. The information propagated from such proteins should be less important compared to the information transmitted from kinases and other proteins differentially expressed and having high disease associations. On that account, we added a propagation attention mechanism to increase the importance of these nodes. Specifically, we implemented a mechanism to learn a dynamic and adaptive summary of the local neighborhood, which operates only in the feature space [64]. The attention from node *i* to node *j, γ_i,j_*, is defined as

$$\begin{array}{cc} \gamma_{i,j}=\frac{e^{\beta \;\cos(x_{i},x_{j})}}{\sum_{k\in N(i)\cup \{i\}}\;e^{\beta \;\cos(x_{i},x_{k})}} & \text{Equation 2} \end{array}$$

where *N* (*i*) denotes the neighbors of node *i* and *β* is a trainable parameter. Essentially, the attention is the softmax of feature cosine similarities between center nodes and their neighbors. By utilizing the attention mechanism, the original propagation matrix calculated from the degree and adjacency matrices shown in Equation 1 can be replaced with a new propagation matrix Γ, which adaptively adjusts propagation weights based on neighbor features. This new propagation scheme addressing the problem of equal weights can be expressed as

$$\begin{array}{cc} {\text{X}}^{\left(\text{t}\right)}=\Gamma X^{\left(\text{t}-1\right)} & \text{Equation 3} \end{array}$$

where each entry of the propagation matrix Γ is calculated using Equation 2.

### Node embeddings

After the information is propagated, the embeddings of each node need to be updated. Many techniques are available to generate node embeddings, and each has its advantages and disadvantages. Based on a series of preliminary experiments, we decided to implement a model inspired by the graph isomorphism network (GIN) [65]. The GIN offers an exceptional performance and has a relatively simple structure, which is important for our model because even after reduction, the cancer input data are much larger than typical datasets used in other fields. Briefly, the GIN transforms the graph isomorphism to the context of deep learning. Nonetheless, it employs a rather basic propagation scheme summing up features from all neighbor nodes. In order to further increase the performance, we replaced this simple propagation step with the attention-based propagation scheme shown in Equation 3. Combining the GIN update protocol with the propagation attention results in a very efficient graph convolution block expressed as

$$\begin{array}{cc} {\text{X}}^{'}=\Theta \left(\left(\Gamma +(1+\varepsilon )\cdot \text{I}\right)\cdot X\right) & \text{Equation 4} \end{array}$$

where Θ denotes a neural network, Γ is the propagation matrix calculated using Equation 2 and ε is a trainable parameter.

### Graph readout mechanism

CancerOmicsNet employs JK-Net followed by a Set2Set model to generate a global representation of the input graph from the node-wise information. JK-Net exploits varying influence radii of different layers to learn the best representation of the entire graph. This model can integrate outputs from individual graph convolutional blocks with three strategies, the concatenation, the max-pooling, and the LSTM attention. Considering the size of our data, we decided to employ the max-pooling strategy since it does not introduce any additional hyperparameters. This particular strategy performs a feature-wise max-pooling with lower layers favoring the local information and higher layers mostly containing the global graph information. With the max-pooling scheme, JK-Net automatically selects the most informative neighborhood size for each feature coordinate. Once the information from different layers is aggregated, we adopted the Set2Set model [43] as a final attention-based readout mechanism. A conventional method to simply flatten all embeddings is unsuitable for orderless graphs, which require a premutation-invariant readout mechanism instead. Set2Set comprises three blocks, a reading block, a process block, and a write block. In CancerOmicsNet, the reading block generating embeddings for each item in the set is replaced by JK-Net aggregating information from multiple graph convolutional blocks. The process block is an LSTM that reads the embeddings and state generated from the previous processing step, and outputs a new hidden state. Finally, the write block is also an LSTM, which takes the hidden state as a context to generate the attention for each item in the set. Subsequently, the attention vector is combined with the embedding matrix using a weighted summation to generate new, permutation-invariant embeddings.

### Other methods to predict cancer drug response

CancerOmicsNet is compared to several other methods to predict the growth rate of cancer cell lines after drug treatment against the same dataset and employing the same cross-validation protocol. The graph isomorphism network (GIN) incorporates the graph isomorphism test to generate node embeddings preserving the original graph structure at each propagation step [65]. As a result, the propagation process contains not only the propagated information, but also the node information in the original graph as an extra term. The Weisfeiler-Lehman (WL) Tree is a widely adopted graph kernel method for graph machine learning [44]. This algorithm utilizes kernel functions and the WL graph isomorphism test to iteratively generate new labels for nodes and new representations for graphs. By iteratively propagating the information, the final information for each node and the entire graph can be extracted.

Cancer Drug Response Profile scan (CDRscan) is a deep learning model predicting drug response from cancer genomic signature [23]. CDRscan employs two input data, the genetic mutation information and the molecular profiles of drugs represented by PaDEL-descriptors [66]. In order to apply CDRscan to our dataset, the mutation information was substituted with the gene expression of cancer cell lines. Following the original implementation, the input data are passed through CNNs to extract features, which are then concatenated to make the final prediction. In the original paper, five slightly different models were employed in order to create an ensemble model. However, since there neither fundamental differences among these models nor a significant performance improvement of the ensemble model, we implemented the best performing single model according to the original benchmarks.
